# Awareness of Placental Pathologic Examination Criteria and Utilization of Pathology Reports among Obstetricians

**DOI:** 10.3390/medicina59030574

**Published:** 2023-03-15

**Authors:** Amal AlOdaini, Ghaida AlKhalifah, Lina Alafghani, Nawal Bin Jalalah, Norah Alsuwailem, Zainab AlMomen

**Affiliations:** 1 Pathology Department, King Fahd University Hospital, College of Medicine, Imam Abdulrahman Bin Faisal University, P.O. Box 1982, Dammam 31441, Saudi Arabia; 2 King Fahd University Hospital, College of Medicine, Imam Abdulrahman Bin Faisal University, P.O. Box 1982, Dammam 31441, Saudi Arabia

**Keywords:** CAP guidelines, placenta, pathologic examination, obstetrics

## Abstract

*Background and Objectives*: Several studies have reported a low rate of pathological examination of the placentas and a poor utilization of pathology reports. We assessed Saudi obstetricians’ awareness and utilization of the placental pathological examination guidelines of the College of American Pathologists (CAP) and evaluated their understanding of the reports. *Materials and Methods*: An anonymous survey was distributed to obstetricians registered in the Saudi Commission of Health Specialties database. We examined the association between the participants’ level of training or practice as well as their institution type with the surveyed elements. *Results:* Of 292 respondents, 34.2% were aware of the CAP guidelines. Most of them were practicing in government hospitals. Moreover, 18.2% of them routinely sent the placenta for pathological examination, and approximately 70.5% routinely reviewed the pathology reports and understood the nomenclature used; these percentages were significantly higher among university hospital practitioners. The residents were the least aware of the CAP guidelines and the least likely to review and understand the pathology reports. Regardless of the CAP guidelines awareness, the most common indication for placental pathologic examination was fetal anomalies, followed by medicolegal reasons and infections. *Conclusions*: Placental pathologic examination appeared uniformly underutilized in Saudi Arabia. Obstetricians are required to generate awareness of the need to comply with the CAP guidelines and to improve the understanding and utilization of pathology reports.

## 1. Introduction

In many aspects, the placental pathologic examination differs from the standard surgical pathology specimen examination. Placental pathology reflects pathology from three distinct sources: (1) the fetal compartment because the placenta is the largest fetal organ; (2) the maternal compartment because the placenta is housed in and perfused by the mother; and (3) organ-specific abnormalities intrinsic to the placenta itself. All three of these elements are pertinent to the obstetric and pediatric. The histopathological examination of placentas has a substantial clinical utility. In addition to its role in understanding the pathophysiology of pregnancy, it facilitates the understanding of adverse fetal outcomes and various pregnancy complications such as hypoxic brain injury, fetal growth restriction, premature delivery, and neonatal neurological injury [1,2]. Placental examination is especially important in cases of stillbirth to help reduce the proportion of “unexplained” circumstances. A diligent and meticulous placental examination reduces the proportion of “unascertained” stillbirths by 83% [1]. Furthermore, in a study of 1025 perinatal deaths, placental examination was shown to be more valuable than autopsy [2]. Placental pathologic examination improves the management of future pregnancies and assists in the medicolegal evaluation of an adverse pregnancy outcome [3,4]. The placenta can also be a good source of tissue for chromosome analysis, especially during fetal death when the fetus is macerated.

Conventionally, all placentas should undergo a gross examination either by a pathologist or by the delivering clinician/care giver. Considering that not all placentas can undergo a full pathologic examination due to costs, resources, and personnel constraints, the selection of placentas for pathologic examination should be based on validated indications that have the potential to provide insights into both the mother and the infant’s immediate and long-term outcomes, warn about risks for future pregnancies, and add value to the practice of obstetrics, neonatology, and clinical genetics. In 1997, the Placental Pathology Practice Guideline Development Task Force of the College of American Pathologists (CAP) published comprehensive and simple guidelines that include maternal, fetal, and placental indications to determine when an obstetrician or healthcare workers involved in deliveries are recommended to select and send the placenta for full histopathologic examination (Table 1) [3,5,6,7]. In summary, the maternal factors are classified into several subgroups. The first is a previous history of reproductive loss, which includes preterm birth, spontaneous abortion, stillbirth, or neonatal death. The second group of indications involves the maternal medical history, such as a personal history of hypertension, coagulopathy, or diabetes, and the final group of indications involves the current pregnancy. This includes virtually any pregnancy or delivery complication, such as prematurity, oligohydramnios, infection, or bleeding. The fetal and neonatal indications are straightforward in that they include any infant problem such as stillbirth, growth restriction, low Apgar scores, anomalies, evidence of fetal distress, and so on. Any abnormality found in the delivered placenta is considered a placental indication. The CAP guidelines recommend that these indications be used on a regular basis. If these guidelines are followed, there is a very slim chance that a placenta with any significant pathology will not be examined. The CAP guidelines creators also defined protocols for the pathologic examination of the placenta, reporting templates for obtained findings and clinicopathologic correlations when applicable [7].

Despite these guidelines, several studies have shown that only a small proportion of the placentas are examined [5,8]. It was shown that up to 20–65% of the placentas meeting examination requirements are not submitted for examination [1,5,8]. The number of submitted placentas for pathological examination continues to decrease despite the specimen’s availability, the moderate costs of a routine pathological examination, and the clear indications [1]. This could be, in part, due to obstetrician’s unawareness of the indications for pathologic assessment of the placenta, as one survey of 111 obstetricians showed that more than 60% of the participants did not know about the CAP guidelines for placenta submission [6]. Financial pressures on the hospital were a cause in other instances [9]. Another possible reason for the dropping number of placenta submissions is the complexity of the reports and misunderstandings between placental pathologists and clinicians about the findings and value of a pathology report [9]. A study showed that the reports are often misinterpreted by the obstetricians due to the nomenclatures used by the pathologists, which are unfamiliar to the obstetricians. This is probably due to the lack of a standardized reporting language among pathologists that would facilitate the communication with obstetricians and maximize its benefits. A report disclosed that clinicians with fewer than 5 years of experience were less likely to read placental pathology reports than experienced clinicians [2,6]. Systematic reviews and several reports have suggested that the obstetricians poorly understand the indications for placenta examination and the findings in the placental pathology report [2,6].

The evidence regarding these issues in Saudi Arabia is limited. One national study in a teaching/tertiary care hospital retrospectively reviewed all placentas submissions for histopathological examination for all deliveries for two years to determine their suitability for pathologic examination. The study found that 16.2% of the delivered placentas during that period met the CAP guidelines; however, only 40.4% of them were sent for pathologic examination. This finding corroborates the international literature and highlights the need to determine the awareness level of the care givers of this topic and the causes of this underestimation, so that corrective measures can be implemented. Therefore, we investigated the nation-wide awareness of the CAP guidelines, the understanding of the nomenclature of placental pathology reports, and the perceived reports’ clinical utility among obstetricians of different experience levels and working environments.

## 2. Materials and Methods

### 2.1. Study Subjects

The tested group was composed of obstetricians; it included consultants, specialists, and residents working at government (public) and university hospitals who were registered in the Saudi Commission for Health Specialties (SCFHS) database which includes 6563 SCFHS-registered obstetricians. The minimum required sample size for the quantitative survey was calculated to be 260 participants (Epi Info^®^ software version 7.0, US Centre for Disease Control (CDC) statistical software, Atlanta, GA, USA) on the assumption that 50% of the obstetricians were aware of the CAP guidelines to submit a placenta and they understood the nomenclature in the pathological report, at a confidence level of 95%, and with a precision of 5.

### 2.2. Study Materials

This was a non-experimental cross-sectional study based on a structured questionnaire survey. We adopted and modified the survey tool of the previous survey by Odibo et al. [6] and converted it to an online survey using Google Forms. The survey comprised three sections. The first section included the statement of the study aim, the informed consent form, and the data confidentiality agreement. The second section addressed the demographic questions, including the level of training (resident or consultant/specialist), the number of years in practice, and the institution type where the participant worked (e.g., government or university hospital). The third section included 10 close-ended survey questions to assess the participant’s awareness of the CAP guidelines, the indications for placental examination in the absence of CAP guidelines awareness, and the utilization of the findings obtained from the pathology reports. Using a convenient sampling technique, anonymous surveys were sent by the SCFHS, on our behalf, to obstetricians registered in their database from 1 November 2020 to 1 January 2021, through institutional or personal e-mails, along with a weekly reminder to fill out the survey.

### 2.3. Statistical Analysis

Descriptive statistics were used to summarize the demographic characteristics of the participants and the surveyed variables. Statistical analyses were performed using the Statistical Package for Social Science software (IBM SPSS Statistics for Windows, Version 22.0., Armonk, NY, USA: IBM Corp.). Chi-square tests or Fisher’s exact tests, as appropriate, were employed to assess the association between the three obstetrician groups (residents, consultants or specialists with <15 years of practice, and consultants or specialists with ≥15 years of practice), different institutions (government or university hospital), and all the survey questions. All analyses were based on two-sided tests, with a *p*-value of <0.05 considered statistically significant. Multivariable logistic regression analyses were applied to explore the factors affecting the CAP guidelines’ awareness and utility. The odds ratio was interpreted as the probability of sending the placenta for pathologic examination, of being aware of the CAP guidelines, and of considering the CAP guidelines as applicable, while the three variables examined were the level of training, the years of experience, and the type of the participants’ institution.

## 3. Results

### 3.1. Demographic Characteristics of the Participants and Their Attitude toward Pathologic Examination

Out of the 6563 distributed surveys, 292 completed surveys were received. Most of the participants were consultants and specialists with ≥15 years of practice, followed by consultants and specialists with <15 years, and residents (48.2%, 25.6%, and 26%, respectively). Slightly over half of the participants had less than 15 years of work experience, and the majority of them worked in government (public) hospitals (Table 2).

Only 53 participants routinely sent the placentas for pathologic examination (18.2%), a number that was slightly higher among those working in government hospitals, but the difference was not statistically significant. About three-quarters of the respondents reviewed the pathology reports routinely. They understood the terminology used in the report (Table 3), which was significantly more frequent in respondents who practiced in university hospitals (Table 4). Most of the participants mentioned that the pathology reports were useful (90.1%) and they would continue to send placentas for pathologic examination (87.0%).

Moreover, 183 participants believed that the placental pathologic examination had been useful in medicolegal settings during their practice (62.7%); this number was higher among the obstetricians working in government hospitals (63.1%) but did not differ across the three practicing groups (Table 5). Only 100 respondents (34.2%) were aware of the CAP placental pathologic examination guidelines, mainly the practitioners working in government hospitals. However, almost 81% of the participants thought that these guidelines were clinically helpful (Table 3).

Another statistically significant difference was the one concerning the rate of routinely reviewing the placenta pathology reports and the understanding of the report’s nomenclature. The consultants and specialists with less than 15 years of experience were more likely to routinely review and understand the nomenclature of the report, followed by the consultants and specialists with more than 15 years of experience, and lastly, the residents (84.0%, 78.7%, 48.6%, respectively, with *p*-values of 0.002 and <0.001). The respective percentages were higher among responders who were working in university-based hospitals.

Higher rates of residents and consultants/specialists with less than 15 years of experience reported the usefulness of the pathology reports compared to those of participants with more than 15 years of experience (*p* = 0.049). Regardless of their years of experience and host institution, most of the participants declared that they would continue to send placentas for pathologic examination (Table 5).

### 3.2. Clinical Indications Used by the Participants for Pathological Placental Examination When the CAP Guidelines Were Not Used

**Figure 1:**
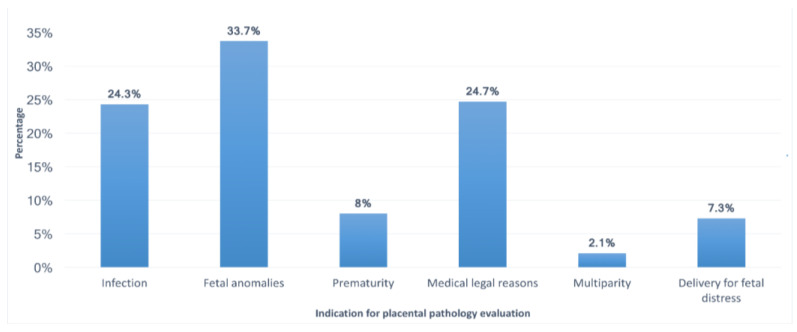
Indications for pathological examination of placentas when the CAP guidelines were not used.

When the CAP guidelines were not used, fetal anomalies were the most common indication to send placentas for pathologic examination, among the three subgroups of participants (Figure 1).

**Figure 2:**
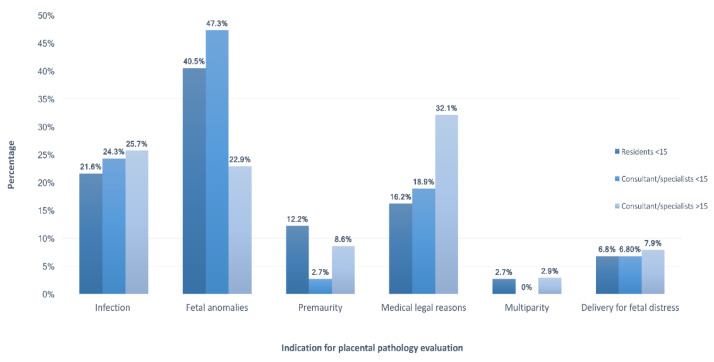
Indications for pathological examination of placentas in relation to different levels of the participants’ training and experience when the CAP guidelines were not used.

**Figure 3:**
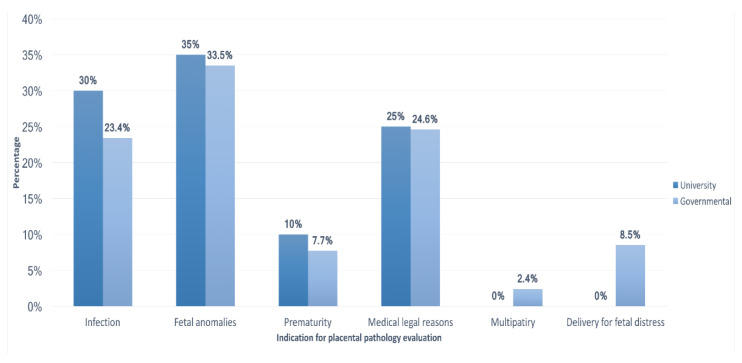
Indication for pathological examination of placentas in relation to different institutions when the CAP guidelines were not used.

Moreover, fetal anomalies were the most common indication for sending placentas for pathologic examination among respondents working in both types of hospitals. Medicolegal reasons were the most common indication among consultants or specialists with more than 15 years of experience (32.1%), while fetal anomalies were the most common indication among those with less than 15 years of experience (47.3%) and among residents (40.5%). The least frequent reason used by all participants to send the placenta for pathologic examination was multiparty regardless of their institutions’ type. The variations between these indications were significant when comparing participants with different levels of training and years of experience (*p* = 0.01; Figure 2). However, no significant difference was observed with respect to the participants’ institutions (*p* = 0.388; Figure 3).

When comparing the three groups of obstetricians (residents, consultants or specialists with less than 15 years of experience, and consultants or specialists with more than 15 years of experience), the rates of those routinely sending placentas and being aware of the CAP guidelines were found to be approximately similar (Table 5). In contrast, there was a significant difference among the participants in their appreciation of the clinical usefulness of the CAP guidelines, which was the highest among the consultants and specialists with less than 15 years of experience.

The multivariate analysis (Table 6) revealed that the odds of CAP guidelines awareness for the consultants or specialists were 0.866 times higher than the odds of the awareness of the residents. Similarly, the odds of sending the placenta for pathologic examination and appreciating the usefulness of the CAP guidelines were, respectively, 0.445 and 2.920 times those applicable for residents. Similarly, for the respondents with more than 15 years of experience, the odds of the awareness of the CAP guidelines and of sending a placenta for pathologic examination were 0.564 and 0.529 times higher than the odds applicable for those with less than 15 years of experience. Moreover, the odds of appreciating the usefulness of the CAP guidelines were 1.512 times higher than the odds applicable for those with less than 15 years of experience. Finally, the odds of the awareness of the CAP guidelines, sending a placenta for pathologic examination, and appreciating the usefulness of the CAP guidelines among the obstetricians and gynecologists practicing in government hospitals were, respectively, 0.811, 0.562, and 1.773 times the odds applicable for those practicing at the university hospitals.

## 4. Discussion

A healthy placenta is essential for fetal survival as well as for ensuring maternal adaptation to pregnancy. Challenges in real-time pregnancy assessment due to the placenta’s inaccessible location have hindered studies, and thus the placenta is known as the “least comprehended” human organ. A careful examination of the placenta, along with a microscopic examination, is a valuable tool of great clinical importance that can quite often shed light on many risk factors and on the pathogenesis of adverse maternal, neonatal, and fetal events. Furthermore, the examination of the placenta aids in the prevention of these events as well as in the provision of treatment that can be offered in future pregnancies. Placental pathologic examination aids as well in the resolution of medico-legal issues in malpractice cases. Because many placentas are normal, the examination of all placentas may not be warranted and may be impractical due to time and resource constraints, particularly in hospitals with a high volume of deliveries. The CAP guidelines with recommendations on sending a placenta for pathologic examination were developed by a multidisciplinary group of pathologists, maternal–fetal medicine specialists, and neonatologists to aid the obstetricians’ decision making [7]. These guidelines are intended to be a standardized approach to be used when certain maternal, fetal, and placental conditions indicate the need for a pathologist to interpret the placenta grossly and microscopically. The sensitivity and specificity of the pathologic examination of the placenta in conformity to the CAP guidelines were 63.4% and 91.6%, respectively [10]. Despite the clarity and practicality of these guidelines, many obstetricians are either unaware of them or not adhering to them. This conclusion can be drawn by considering the falling number of placentas submitted to pathologic examination. For example, Aljhdali et al. studied the practice of placenta submission for histopathological examination in a teaching/tertiary Hospital in Saudi Arabia; out of 8929 deliveries, 1444 (16.2%) placentas met the CAP guidelines, and only 583/1444 placentas (40.4%) were sent for pathologic examination [9]. Similar findings were reported in Aysha and Rafaat’s 2020 retrospective study in the United States, which indicated that 213 (42.6%) of 500 placentas should have been submitted for pathology evaluation, but only 135 (27% of the total) were submitted [10]. These studies and others showed a significant number of placentas that were not submitted for pathological studies, even though they met the CAP criteria [9]. These observations were the basis of our study which aimed to uncover the CAP guidelines understanding status among obstetricians of different training/experience levels who practice in different clinical settings. In our study, most participants perceived the utility of the pathology report and were encouraged to continue to send placentas for examination, regardless of their level of training or years of experience. However, only 34.4% of them were aware of the CAP guidelines, reflecting the possibility of inappropriate request for pathologic examination and their poor knowledge of the indications for placenta examination due to possible inadequate training or an outdated curriculum. Surprisingly, 80.9% of our respondents found the CAP guidelines clinically useful. The discrepancy between not knowing the guidelines and finding them clinically useful might be due to the general belief that all guidelines help in clinical decisions and eliminate a vague practice. In agreement with our results, only 36% of the Odibo et al. [6] study participants were aware of the CAP guidelines. In that study, an increased level of awareness of the CAP guidelines was associated with higher levels of experience [6]; in contrast, our study revealed an indifferently low awareness of the CAP guidelines regardless of the level of training, the years of experience, or the host institution’s type of the participants. As the CAP guidelines are very detailed, our participants might have been aware of the main categories in the guidelines but were unaware of the sub-categories. Using different guidelines or methods (such as gross examination) to determine the need to send a placenta might also explain the low awareness of the CAP guidelines. Regardless of the CAP guidelines, most obstetricians in our study requested a placental pathologic examination for fetal anomalies. In contrast, most obstetricians in the Odibo et al. [6] study requested a placental pathologic examination for infections. A variation in the prevalence of infections and fetal anomalies might have influenced these ratios. A study showed that placentas were more commonly examined for fetal indications than for maternal ones [3]. However, in another study, the maternal indications were the most common for a placental pathological examination [10]. The study of Booth et al. [11] showed that the maternal and fetal conditions suggested by the CAP guidelines were the least likely reasons to send a placenta for examination, compared to surgical delivery and low birth weight. Gestational age, mode of delivery, infant admission to the neonatal intensive care unit, maternal fever, and gross placental abnormalities were also reported as factors affecting the obstetricians’ decision to send a placenta for pathologic examination [5]. It is unclear if the decision was based on a hospital policy or on the obstetricians’ clinical judgment. In our study, there was no difference in the rates of sending placentas for examination in the different institutions, an observation that was not in agreement with that of the Odibo et al. [6] study, where the obstetricians working in community hospitals used to send the placentas for a pathologic examination more frequently. The low number of participants from university hospitals might have falsely influenced our results. As mentioned earlier, a lack of awareness of the CAP guidelines was evident in all institutions, explaining the consistently low rates of sending placentas for a pathologic examination.

The placental reports are supposed to provide the clinician with enough information to counsel the parents and clarify the possible pathophysiological pathways that gave rise to an adverse outcome, the risks of recurrence, and the treatment options available in future conceptions. Presently, the standard practice for placental pathology is narrative reporting, which is prone to bias and quality issues. As a result, the quality of placental reporting is known to vary significantly, ranging from extremely simplistic to extremely detailed, with a high level of variations in the reported findings. This may complicate the obstetrician’s understanding and defy the purpose of the report. This has at times even caused clinicians to misunderstand pathologists’ reports, resulting in medical errors [12]. This is particularly true for less experienced practitioners and residents. While one study showed no difference in the understanding of the report’s nomenclature between obstetricians with different levels of training [6], we found that higher levels of training and working experience in a university hospital were associated with routinely reviewing pathology reports and having a good understanding of the report’s nomenclature. A similar observation by Walsh et al. was reported, indicating that experienced clinicians read placental pathology reports at a significantly higher rate (46/47 [97.9%]) than less experienced clinicians (11/15 [73.3%]; *p* = 0.01) [2]. Contrary to our hypothesis, which suggested a poor understanding of the pathology report’s nomenclature by the majority of the obstetricians, only 27.4% of our participants declared that they had difficulties understanding the pathology report. More than half of the consultants and specialists could understand the pathology report’s terminology; however, the residents were the least likely to review the pathology reports, and only half of them could understand them. These observations make it crucial to explore the possible reasons for these knowledge gaps between obstetricians working in different institutions and between those with different experiences. One of the possibilities is inadequate knowledge delivery during the training years. Poor communication between obstetricians and pathologists in government hospitals, which might have influenced their understanding, is another potential cause. The obstetricians are encouraged to consult with pathologists about placental reports, particularly if there is a disparity between the clinical evaluation and the reported diagnoses. Finally, the use of synoptic pathology reports with a standardized diagnostic nomenclature, rather than the narrative one, would improve the understanding of the report and its benefits, as a survey among obstetricians on the utility of the placental pathology reports found that a more streamlined report, with findings organized by lesion category, outperformed the narrative reporting format in terms of improving the interpretation and implementation of the findings into clinical practice [2].

An obvious limitation of our study is the small sample size. Despite the surveys being distributed to more than 6000 obstetricians, only 292 obstetricians responded (4.4% of the target population). Moreover, our sample included an extremely low number of obstetricians working in university hospitals and no participants from private hospitals. We are aware that despite the important findings, our results cannot be generalized. Therefore, further studies should be conducted to include a more significant number of obstetricians working in universities and private hospitals.

## 5. Conclusions

The placenta has long been overlooked, with little understanding of the importance of its examination. This study produced essential results that show the importance of raising the awareness about the CAP guidelines among obstetricians to bridge the practitioners’ knowledge gap in hope of maximizing the utility of the placental pathologic report. The findings, also, highlight the importance of clinician–pathologist cooperation in this area, which will most likely lead to improvements in the level of obstetrics and neonatal care. Finally, to increase the overall utility of placental pathology, a standardized reporting approach in nomenclature and diagnostic classification is advised. Due to the small sample size, further studies should be conducted to include more obstetricians working in universities and private hospitals so to reach a more solid conclusion of the current situation, allowing each institution to propose realistic policies and develop its own set of guidelines based on the CAP guidelines and tailored to its population.

## Figures and Tables

**Table 1:** Clinical indications for the pathologic examination of the placenta, as set by the CAP 1997 Guidelines.

Maternal indications	Unexplained/recurrent pregnancy complication: IUGR, spontaneous abortions, stillbirths, or premature births Systemic disorder: coagulopathy, severe diabetes mellitus, impaired glucose metabolism, hypertension (preeclampsia, pregnancy-induced or chronic), collagen disease, seizure, severe anemia (<9 g/dL) Prematurity (≤34 weeks) Postmaturity (>42 weeks) Severe oligohydramnios Severe unexplained polyhydramnios Fever or infection (e.g., HIV, syphilis, CMV, primary herpes, toxoplasma, rubella) Unexplained 3rd trimester bleeding or excessive bleeding >500 mL Invasive procedure with suspected placental injury Abruptio placentaeNon-elective pregnancy terminationThick or viscid meconium
Fetal indications	Admission or transfer to other than level 1 nursery Stillbirth or perinatal death Compromised clinical condition such as any of the following: cord blood pH < 7.0; Apgar score ≤ 6 at 5 min; ventilatory support, >10 min; severe anemia, hematocrit < 35% Seizure Infection or sepsis Major congenital anomalies, dysmorphic phenotype, or abnormal karyotype Discordant twin growth, more than 20% weight difference Multiple gestation with same-sex infants and fused placentas Birth weight > 95th percentile Asymmetric growth Multiple gestation without other indication Vanishing twin beyond the 1st trimester
Placental indications	Physical abnormality (e.g., infarct, mass, vascular thrombosis, retroplacental hematoma, amnion nodosum, abnormal coloration, or opacification, malodor) Small or large placental size or weight for gestational age Umbilical cord lesions (e.g., thrombosis, torsion, true knot, single artery, absence of Wharton’s jelly) Total umbilical cord length less than 32 cm at term Abnormalities of placental shape Long cord (more than 100 cm) Marginal or velamentous cord insertion

Abbreviations: CAP, College of American Pathologists; CMV, cytomegalovirus; HIV, human immunodeficiency virus; IUGR, intrauterine growth restriction.

**Table 2:** Demographic characteristics of the participants.

Study Demographics		Statistics, N (%)
Level of training	Residents <15 years	76 (26%)
	Consultants/specialists <15 years	75 (25.6%)
	Consultants/specialists ≥15 years	141 (48.2%)
Years of practice	<15	151 (51.7%)
	≥15	141 (48.2%)
Institution	Government hospital	252 (86.3%)
	University hospital	40 (13.7%)

**Table 3:** Descriptive statistic of the pathologic examination survey questions.

**Survey Questions**		**Statistics, N (%)**
Do you routinely send placenta for pathologic examination?	No	239 (81.8%)
	Yes	53 (18.2%)
Are you aware of the CAP guidelines for placental pathology evaluation?	No	192 (65.7%)
	Yes	100 (34.2%)
Given an awareness of the CAP guidelines, are they clinically useful?	No	56 (19.1%)
	Yes	236 (80.9%)
If the CAP guidelines are not used, how do you determine the need for pathology evaluation?	Infection	72 (24.6%)
	Fetal anomalies	99 (33.9%)
	Prematurity	23 (7.9%)
	Medicolegal reasons	71 (24.3%)
	Multiparty	6 (2.1%)
	Delivery for fetal distress	21 (7.2%)
Do you routinely review pathology reports?	No	86 (29.5%)
	Yes	206 (70.5%)
If you routinely review reports, do you understand the nomenclature of the report?	No	81 (27.7%)
	Yes	211 (72.3%)
Are the pathology reports useful?	No	29 (9.9%)
	Yes	263 (90.1%)
Has the result of a placenta examination ever been useful to you in a medicolegal situation?	No	109 (37.3%)
	Yes	183 (62.7%)
Should we continue to send placentas for pathological evaluation?	No	38 (13.0%)
	Yes	254 (87.0%)

**Table 4:** Bivariate analysis comparing the responses to the survey questions across different institutions.

Survey Questions		Government Hospital	University Hospital	*p*-Value
Do you routinely send placenta for pathologic examination?	No	205 (81.3%)	34 (85%)	0.387
	Yes	47 (18.6%)	6 (15%)	
Are you aware of the CAP guidelines for placental pathology evaluation?	No	163 (64.6%)	29 (72.5%)	0.211
	Yes	89 (35.3%)	11 (27.5%)	
Given an awareness of the CAP guidelines, are they clinically useful?	No	45 (17.8%)	11 (27.5%)	0.11
	Yes	207 (82.1%)	29 (72.5%)	
Do you routinely review pathology reports?	No	85 (33.7%)	1 (2.5%)	0.000
	Yes	167 (66.2%)	39 (97.5%)	
If you routinely review reports, do you understand the nomenclature of the report?	No	80 (31.7%)	1 (2.5%)	0.000
	Yes	172 (68.2%)	39 (97.5%)	
Are the pathology reports useful?	No	28 (11.1%)	1 (2.5%)	0.064
	Yes	224 (88.8%)	39 (97.5%)	
Has the result of a placenta examination ever been useful to you in a medicolegal situation?	No	91 (36.1%)	18 (45%)	0.189
	Yes	161 (63.1%)	22 (55%)	
Should we continue to send placentas for pathological evaluation?	No	34 (13.1%)	4 (10%)	0.389
	Yes	218 (86.9%)	36 (90%)	

**Table 5:** Bivariate analysis comparing the responses to the survey questions across the three groups of obstetricians.

Survey Questions	Consultant/Specialist with ≥15 Years of Experience	Consultant/Specialist with <15 Years of Experience	Resident (<15 Years of Experience)	*p*-Value	
Do you routinely send placenta for pathologic examination?	No	116 (82.2%)	60 (81.0%)	63 (82.8%)	0.974
	Yes	25 (17.7%)	15 (20%)	13 (17.1%)	
Are you aware of the CAP guidelines for placental pathology evaluation?	No	87 (61.7%)	48 (64%)	57 (75%)	0.184
	Yes	54 (38.3%)	27 (36.0%)	19 (25.0%)	
Given an awareness of the CAP guidelines, are they clinically useful?	No	25 (17.7%)	9 (12.0%)	22 (28.9%)	0.037
	Yes	116 (82.2%)	66 (88%)	54 (71.1%)	
Do you routinely review pathology reports?	No	34 (24.1%)	17 (22.6%)	35 (46.1%)	0.002
	Yes	107 (75.8%)	58 (77.3%)	41 (53.9%)	
If you routinely review reports, do you understand the nomenclature of the report?	No	30 (21.2%)	12 (16.0%)	39 (53.9%)	0.000
	Yes	111 (78.7%)	62 (84.0%)	37 (48.6%)	
Are the pathology reports useful?	No	20 (14.1%)	3 (4.0%)	6 (7.8%)	0.049
	Yes	121 (85.8%)	72 (96%)	70 (92.1%)	
Has the result of a placenta examination ever been useful to you in a medicolegal situation?	No	48 (34.0%)	27 (36.1%)	34 (44.7%)	0.326
	Yes	93 (65.9%)	48 (64%)	42 (55.1%)	
Should we continue to send placentas for pathological evaluation?	No	21 (14.8%)	5 (6.7%)	12 (15.7%)	0.192
	Yes	120 (85.1%)	70 (93.3%)	64 (85.2%)	

**Table 6:** Multinomial logistic regression.

Variables	Odds Ratio (95% Confidence Interval), Estimate							
	Aware of the CAP Guidelines	Send Placenta	Usefulness of CAP Guidelines	Nomenclature of Report	Review Pathology Reports	Pathology Reports Are Useful	Placenta Examination Usefulness	Continue to Send Placentas
Level of experience (Ref: resident)	0.866 (0.536–1.399), −0.144	0.445 (0.256–0.773), −0.810	2.920 (1.669–5.107), 1.071	10.674 (5.368–21.222), 2.368	6.313 (3.450–11.553), 1.843	8.338 (2.926–23.764), 2.121	1.420 (0.894–2.256), 0.351	4.881 (2.318–10.274), 1.585
Years of experience (Less than 15 years)	0.564 (0.350–0.907), −0.573	0.529 (0.298–0.941), −0.636	1.512 (0.841–2.719), 0.413	2.581 (1.341–4.967), 0.948	2.166 (1.209–3.881), 0.773	13.330 (4.276–41.551), 2.590	0.897 (0.563–1.430), −0.109	4.241 (1.935–9.297), 1.445
Institution (university vs. government hospital)	0.811 (0.471–1.398), −0.209	0.562 (0.300–1.055), −0.575	1.773 (0.933–3.369), 0.573	0.259 (0.122–0.549), −1.350	0.362 (0.185–0.708), −1.016	0.610 (0.200–1.860), −0.494	1.454 (0.854–2.478), 0.375	1.077 (0.474–2.447), 0.74

## Data Availability

The data presented in this study are available in the article.
